# The chromosome complement of male *Chrysoperla rufilabris* (Burmeister, 1839), with evidence of distance segregation in meiosis I

**DOI:** 10.17912/micropub.biology.001770

**Published:** 2025-09-17

**Authors:** Valoria Maslin, Allison Eisenhauer, Leocadia Paliulis

**Affiliations:** 1 Biology Department, Bucknell University, Lewisburg, Pennsylvania, United States

## Abstract

Neuropteran insects display a phenomenon called distance segregation during male meiosis I, where the X and Y chromosomes are unconnected from prometaphase I through anaphase I. Here we investigate chromosome number and patterns of chromosome segregation of a previously unstudied Neuropteran: the green lacewing, *Chrysoperla rufilabris* (Burmeister, 1839). Testes of male *C. rufilabris* were isolated and prepared following a chromosome squash method. Living meiosis I spermatocytes were also imaged. The data revealed a chromosome number of 2n=12 in males (2n=10+XY) and evidence of distance segregation of X and Y chromosomes in meiosis I spermatocytes.

**Figure 1. The chromosomes of Chrysoperla rufilabris males:**
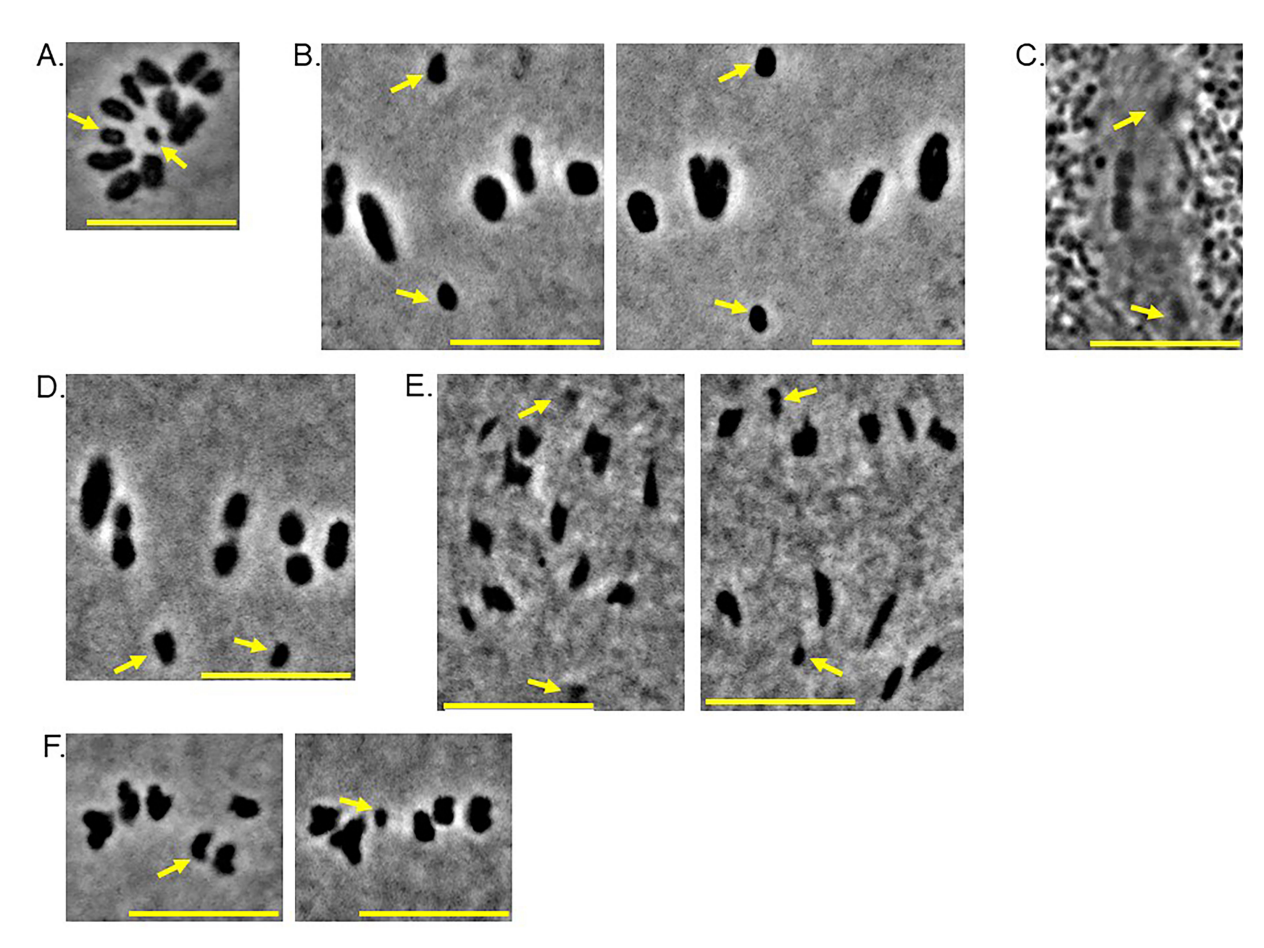
(A) Squash preparation of a spermatogonium in mitosis from the green lacewing *Chrysoperla rufilabris* . There are twelve chromosomes, indicating a diploid chromosome number of 2n=12. Arrows show X and Y chromosomes. 12 squashed mitotically-dividing spermatogonia were imaged and included in the dataset for this project. Bar=10 μm. (B) Representative images of squash preparations of meiosis I spermatocytes. Five autosomal bivalents are visible, aligned on a metaphase plate, in addition to the distance segregating sex chromosomes (arrows). 55 squashed meiosis I spermatocytes were imaged and included in the dataset for this project. Bar=10 μm. (C) Living metaphase I spermatocyte. Sex chromosomes shown by arrows. Timelapses of five different living cells were obtained and are included in the analysis of these data. Bar=10 μm. (D) Squash preparation of a meiosis I spermatocyte with incorrectly-oriented sex chromosomes (arrows); i.e. sex chromosomes associated with the same spindle pole. Of the 55 squashed meiosis I cells studied, only one had both sex chromosomes facing the same pole. Because this is a fixed, stained specimen, it is impossible to know whether this error would be permanent or repaired during the division process. Bar=10 μm. (E) Squash preparations of early (left) and late (right) anaphase I cells. Arrows show sex chromosomes. Bar=10 μm. (F) Squash preparations of meiosis II spermatocytes. Arrows point to the two different sex chromosomes. Each meiosis II cell has six chromosomes and the two cells shown each had a different sex chromosome. Bar=10 μm.

## Description

Distance segregation is a term introduced by Sally Hughes-Schrader to describe chromosomes that move away from each other without being visibly connected beforehand—a special class of achiasmate chromosome segregation (Hughes-Schrader, 1969; Brady and Paliulis, 2015). Previous research indicated that insects in the order Neuroptera have distance-segregating univalent X and Y chromosomes (Naville and de Beaumont, 1933; Naville and de Beaumont, 1936; Klingstedt, 1937; Hughes-Schrader, 1969; Hughes-Schrader, 1979; Hughes-Schrader, 1983; Brady and Paliulis, 2015). The Neuropteran *Chrysoperla rufilabris* (Burmeister, 1839) has not previously been studied chromosomally, is readily available in bulk from several companies that sell insects to support Integrated Pest Management programs, and provided the model for this study of chromosome behavior and distance segregation.

To determine the chromosome number and sex determination system for *C. rufilabris* , fixed, stained testes were prepared using an aceto-orcein squash method. The diploid complement of *C. rufilabris* males, determined by counting chromosomes derived from squash preparations of spermatogonial mitosis (12 cells), is comprised of 12 chromosomes—five pairs of autosomes plus X and Y sex chromosomes ( Figure 1A ). The number of chromosomes and chromosome-based sex determination system was confirmed through observation of squash preparations of dividing cells in meiosis I (55 cells). Examination of meiosis I cells also revealed that X and Y chromosomes segregated early to opposite sides of the cell in metaphase while all autosomes were aligned in a metaphase chromosome configuration ( Figure 1B ). This separation of X and Y chromosomes while autosomes remain connected as a bivalent is indicative of distance segregation of sex chromosomes. Because living cells can, at times, display subtle differences from fixed, stained preparations that have been squashed, we also imaged living meiosis I spermatocytes (5 cells). The sex chromosomes were positioned adjacent to opposite spindle poles while the undivided autosomes aligned at the spindle midline, confirming that distance segregation is present in living cells. In this cell, both sex chromosomes were not centrally located directly on the spindle axis, but were on to the right of the pole-pole axis ( Figure 1C ). This separation of the sex chromosomes in meiosis I, with each on opposite sides of the aligned autosomes was observed in nearly all meiosis I squashes. One cell of the 55 observed, however, had both sex chromosomes on the same side of the metaphase-aligned autosomal bivalents, presumably associating with the same spindle pole—an erroneous alignment ( Figure 1D ). The sex chromosomes in all other observed cells remain at opposite sides of the cell during early and late anaphase I ( Figure 1E ). Chromosome number and sex-chromosome complement were, in addition, confirmed by imaging meiosis II spermatocytes (22 cells). All squashed meiosis II spermatocytes observed had 6 chromosomes ( Figure 1F ). Meiosis II spermatocytes had either a single larger sex chromosome or a single smaller sex chromosome ( Figure 1F, arrows). All raw data that contributed to this analysis are available on Dryad (Paliulis et al., 2025).

This study is the first report of chromosome number, sex chromosome arrangement, and distance segregation in *Chrysoperla rufilabris* . The results are consistent with previous observations of chromosome specialization and segregation patterns of other species of green lacewings (Family: Chrysopidae) that reported a chromosome number of 2n=12 with XX-XY sex determination and apparently unconnected X and Y chromosomes in metaphase I spermatocytes of most species studied (Naville and de Beaumont, 1933; Naville and de Beaumont, 1936; Tauber et al., 2009). This study is also the first to show sex chromosome positions consistent with distance segregation in living primary spermatocytes of any Neuropteran. The sex chromosomes are differently sized from one another in males, as can be clearly seen in Figures 1C, 1D, and 1F. We were unable to observe the chromosome complement of living mitotically-dividing cells of female *C. rufilabris* , and can thus only peripherally deduce that the overall chromosome complement of the species is 2n=12 (with XX/XY sex determination) based on comparisons with previously studied Chrysopidae (Naville and de Beaumont, 1933; Naville and de Beaumont, 1936).

Our observations revealed that both living and fixed/stained X and Y chromosomes were separate prior to nuclear envelope breakdown of meiosis I, but could have variable positions on the spindle near the spindle poles. Hughes-Schrader (1983) suggested that a central and isolated unit of the spindle encompasses the univalent sex chromosomes of Neuroptera and facilitates sex-chromosome distance segregation. We do not see evidence of a central unit in *C. rufilabris* . Figure 1B shows that the sex chromosomes in a living primary spermatocyte were positioned near the spindle poles but on the right side of the spindle. The existence of a cell with an erroneous attachment in which both sex chromosomes associate with the same spindle pole suggests that the sex chromosomes are likely not in a sleeve-like internal spindle component, but examination of microtubule distribution will be essential to determining whether there is an isolated spindle component facilitating distance segregation.

This work provides the foundation for future studies of distance segregation in a system that is easily obtainable in bulk. Future work will provide evidence on how distance-segregating chromosomes attach to the spindle, and consistently separate from one another. Based on our observations of *C. rufilabris* sex chromosomes in living and fixed cells (Figure 1; Paliulis et al., 2025), chromosome behavior in this organism varies substantially from other well-studied systems with achiasmate chromosome segregation (such as yeast or flies) (cf. Wolf, 1993; Hughes et al, 2009; Kurdzo and Dawson, 2015). This could provide evidence either of fine, non-elastic, position-sensing physical connections between sex chromosomes or some other form of position communication between sex chromosomes. Chromosome micromanipulation experiments could shed light on whether, and provide clues as to how, distance-segregating chromosomes are able to communicate their position. For example, repositioning one sex chromosome might cause another chromosome to reorient as was observed by Camenzind and Nicklas (1968) in the mole cricket *Gryllotalpa hexadactyla* . Immunofluorescence using a wide variety of probes might provide candidates for possible physical connections such as the titin-containing tethers observed connecting separating half-bivalents in crane fly spermatocytes (Economopoulos et al., 2025), or could reveal isolated compartments that facilitate distance segregation in this system.

## Methods

**Orcein Staining of Chromosome Squashes**

*Chrysoperla rufilabris* testes were removed from the abdomens of adult males. Isolated testes were transferred into a 3:1 ethanol/acetic acid solution for 5 min. After a 30 s rinse in distilled water, testes were transferred into freshly-filtered aceto-orcein stain (2.5 g orcein in 100 mL 45% (v/v) acetic acid) for 5 min and then onto a slide containing 45% acetic acid for testis distribution using laboratory forceps. Testis follicles were left in acetic acid for 30 sec. A coverslip was centered on the sample and uniform pressure was applied to the coverslip. Slides were sealed with clear nail polish and imaged immediately with a Nikon Eclipse TS100 microscope (Nikon Instruments, Tokyo, Japan) equipped with a 100X, 1.25 NA phase-contrast, oil immersion objective and a View4K HD camera (Microscope Central, Feasterville, PA, USA), using InFocus software version x64 (Microscope Central). Adobe Photoshop CC 2023 was used to crop and rotate images.

**Living-Cell Preparations**

*Chrysoperla rufilabris* testes were removed from the abdomens of adult males and submerged into a culture chamber (described in Lin et al., 2018) filled with a thin layer of Kel-F Oil #10 (Ohio Valley Specialty Company, Marietta, Ohio). Spermatocytes were spread on the coverslip of the culture chamber under a thin layer of oil. Live-cell imaging of the spermatocytes during meiosis I was done using a Nikon Eclipse TSS100 microscope (Nikon Instruments, Tokyo, Japan) equipped with a 100X, 1.25 NA phase-contrast, oil immersion objective. Images were collected using either a View 4K HD camera (Microscope Central, Feasterville, PA, USA), using InFocus software version x64 (Microscope Central), or using a Spot camera with the Spot 3.5.7 software. Adobe Photoshop CC 2023 was used to crop and rotate images.

## Reagents

**Table:** 

**Organism**	**Source**
*Chrysoperla rufilabris* Adult	Sound Horticulture (Bellingham, WA, USA), LWA1 https://soundhorticulture.com (accessed on 06/16/2025)

**Table:** 

**Reagent**	**Source**
Ethanol Absolute	Avantor, 89125-186
Orcein Synthetic	Avantor, TS41657-0050
Glacial Acetic Acid	Avantor, 97064-482
Voltalef 10S PCTFE	Atofina, now Arkema (France)
